# Experimental study on the influence of mental fatigue on risk decision-making of miners

**DOI:** 10.1038/s41598-022-14045-9

**Published:** 2022-07-13

**Authors:** Aifang Jia, Xinyue Guo, Shuicheng Tian

**Affiliations:** 1 College of Safety Science and Engineering, Xi’an University of Science and Technology, Xi’an, China; 2 Department of Mining Engineering, Jincheng Institute and Technical College, Jincheng, China; 3 College of Computer and Information Science, Southwest University, Chongqing, China

**Keywords:** Environmental social sciences, Health occupations

## Abstract

Mental fatigue increases risk-taking behavior. Using data collected between June 15 and August 6, 2020, this study investigates the impact of miners’ mental fatigue on risk decision-making to improve risk prevention and prediction abilities, and to reduce the occurrence of coal mine safety accidents. A total of 273 and 33 people participated in the preliminary and formal experiments, respectively. The participants, coal miners, visited a lab thrice to complete the pre-experiment, Balloon Analog Risk Task (BART), and Iowa Gambling Task (IGT). On the BART, mental fatigue displayed a significantly positive association with risk preference. On the IGT, as mental fatigue increased, net scores continuously decreased, while the frequency of making unfavorable decisions and the probability of taking risks increased. The BART value had no or weak correlations with the net score. Results suggest that mental fatigue leads to an increasing propensity to take risks. Therefore, regarding coal mine safety management, further attention is necessary concerning miners’ mental health, addressing mental fatigue, increasing rest time, and reducing night work. Furthermore, reasonable diet, improved working environments, and a positive attitude toward work should be promoted to reduce or eliminate mental fatigue and avoid decision-making errors that could cause accidents.

## Introduction

It has been reported that 97% of mine accidents in China are caused by people^1^. This is largely attributable to decision-making errors resulting from mental fatigue. As coal mines become increasingly mechanized, various kinds of safety technologies are being adopted. Accordingly, accidents that are relatively uncontrollable have become fewer. However, the proportion of coal mine accidents attributable to human error is increasing. Accidents such as the coal and gas outburst in Guanglong Coal Mine on December 17, 2019; the major gas explosion in Pingyao City on November 18, 2019; and the Ningwu County Coal Mine roof collapse on June 19, 2019 are some examples. Therefore, to prevent human-caused accidents in coal mines, it is important to study the impact of miners’ mental fatigue on their risky decision-making.

Grandjean introduced the concept of mental fatigue^2^, which Thiffault et al.^3^ expounded upon further. Mental fatigue can be described as reduced motivation caused by factors such as lethargy and cognitive impairment^4–8^ resulting from heavy mental work, excessive nervous system tension, or long durations of monotonous and tedious work^4,9–13^. Mental fatigue leads to reduced goal-directed attention and flexibility of behavioral responses, as well as an increase in automatic behavior^14^, negatively impacting the training time required to achieve manual dexterity^15^. Mental fatigue also affects attention, making people less able to suppress irrelevant information as well as behavioral responses based on such information, thereby reducing the accuracy of responses^16^. Mental fatigue has greater impacts in specific situations. The literature has shown that it negatively affects endurance performance^17^, increases the perceived need for physical tasks^18^, and hinders football performance^19^, among others. The Balloon Analog Risk Task (BART) and the Iowa Gambling Task (IGT) are important tools for assessing decision-making^20–24^. Research shows that mental fatigue can make risky decision-making more conservative^25–31^.

It should be noted that all of these aforementioned studies employed laboratory-induced mental fatigue and were conducted over the short term. Therefore, how does mental fatigue at work affect risk decision-making? Very little research has been done, particularly on the effects of mental fatigue in miners on risky decision-making. This paper examines mental fatigue and miners’ risk decision-making, adopts a scientific research concept, makes full use of the research theories of related disciplines, including various analytical tools and methods, and preliminarily demonstrates the effect of mental fatigue on miners’ risk decision-making.

## Materials and methods

In this study, the participants visited the training room three times and participated in the pre-experiment, the BART experiment, and the IGT experiment.

During the first visit, 273 participants answered the questionnaire, 33 of whom were selected for the next experiment. They were asked to get enough rest and food the day before the experiment and were not allowed to overeat, drink alcohol, or consume caffeine. During the second visit, they signed the informed consent form, answered three questionnaires, and underwent the BART experiment. After a one-week interval, the participants visited the lab for the third time, and participated in the IGT experiment.

### Participants

#### The pre-experiment participants

On June 15, 2020, 273 employees of a coal mine in Gaoping, Shanxi, who frequently were required to work underground, were invited to participate in this study. All participants provided informed consent, which was obtained following procedures approved by the Research Ethics Committee of the Institute of Safety Management and Risk Control, Xi’an University of Science and Technology (Grant No. XUST2020058006). The coal mine had 575 employees, of which 273 worked underground often or exclusively. Therefore, only 273 people were invited to participate. All 273 questionnaires distributed were retrieved. Thus, 273 valid questionnaires were obtained.

#### The BART and IGT participants

A total of 33 participants were selected for the pre-experiment, which took place in a quiet training room in the Shouyang Coal Mine in Gaoping from July 28 to August 6, 2020. Participants were required to have obtained adequate rest and food the previous day and were not allowed to overeat, drink, or consume caffeine. After arriving in the training room, participants first read and signed the informed consent form. Participants were informed that they would be paid for their participation in the form of 20% of the prize money obtained in the game. Afterward, they completed the questionnaire. Then, the participants attempted the BART and IGT on a Lenovo desktop computer. After completing the task, they received 20% of the reward they earned in the experiment.

All participants had normal or corrected-to-normal vision and reported no history of neurological or psychiatric disorders. The study was approved by the Academic Committee of Xi’an University of Science and Technology (which has the function of managing academic affairs and the right to approve the rationality and ethics of a study’s experimental design). All experiments were performed in accordance with the relevant guidelines, and all participants gave written informed consent prior to the experiment and were reimbursed for the time they spent at the study site.

### Experimental task

#### The pre-experiment

The questionnaire distributed to the participants was derived from the Maslach Burnout Inventory-General Survey (MBI-GS)^32^. The MBI-GS examines emotional exhaustion, low sense of accomplishment, and dehumanization using 22 items rated on a five-point Likert scale from 5 (never occurred) to 1 (always is the case). Using Kalimo et al.’s^33^ calculation method, the higher the score, the more serious the mental fatigue. Cronbach’s alpha was 0.88 in the current study.

#### BART (Balloon Analog Risk Task)

The BART is a computerized decision-making task developed by Lejuez et al.^34,35^ to assess an individual’s propensity to make risky decisions and has been applied in several domestic and international studies in the field of decision-making^36–38^^.^ This study used a self-edited Chinese version of the balloon simulation task.

Participants performed the BART on a Lenovo desktop computer (17-in. screen) from July 28 to 30, 2020, all with informed consent obtained following procedures approved by the Academic Committee of Xi’an University of Science and Technology. Participants were asked to inflate 30 balloons in a computer simulation. A balloon expanded each time it was clicked, with the participant being awarded 0.2 yuan for each click. Each balloon could be inflated for up to 30 clicks but would explode after a randomly chosen fixed number of clicks, from 1 to 30 (this process was computer-automated). If the balloon exploded, the payoff was 0 yuan. Participants had to decide whether to keep inflating a balloon or stop inflating the balloon and take the money they had earned up until that point. To inflate, they pressed the J key, while to collect the money obtained for that balloon, they pressed the F key. Variables measured consisted of the total number of inflations, the total number of balloons that exploded (0–30), and the total number of unexploded balloons. The BART value consisted of the total number of inflated balloons/the number of unexploded balloons; the experiment featured rewards, not punishments. At the end of the BART experiment, participants received 20% of the prize money they earned in the game.

#### IGT (Iowa Gambling Task)

The IGT is a laboratory task that simulates real decision-making situations. It is an evaluation tool developed by Bechara et al.^39^ to measure risky decision-making and has been applied in various studies in the field of decision-making^40,41^.

Participants performed the IGT on a Lenovo desktop computer (17-inch screen) from August 4 to 6, 2020, all with informed consent obtained following procedures approved by the Research Ethics Committee of the Institute of Safety Management and Risk Control, Xi’an University of Science and Technology. Participants were instructed to choose a total of 100 cards from four decks on the computer screen (A, B, C, or D) in each round to earn as much money as possible and reduce losses; some decks were better choices than others. The variables were as follows: advantageous card choices (0–100), unfavorable card choices (0–100), net score (the number of advantageous cards − the number of unfavorable cards), and total revenue earned. The initial capital per participant was 20 yuan, and the task flagged poor choices. If the participant chose a card from deck A, they would receive a profit of − 1 yuan each time, but five cards out of 10 would include a bonus ranging from 1.5 to 3.5 yuan, which would leave them with 12.5 yuan in total. If the participant chose deck B, they would lose 1 dollar each time, but one out of 10 cards would include a 12.5-yuan bonus. In deck C, the participant would receive a profit of − 0.5 yuan each time, but five cards out of 10 would include a bonus of 0.25 to − 0.75 yuan, which would leave them with 2.5 yuan in total. When choosing from deck D, the participant would get − 0.5 yuan each time, but one out of 10 cards would include a bonus of 2.5 yuan. Therefore, in the long run, A and B were the better decks, while C and D reflected poor choices. Before the experiment, participants were unaware of which decks were favorable. They were only told that some of the decks were favorable while some were unfavorable, and that they had to choose favorable cards to obtain the maximum profit.

#### Questionnaire

As risk decision-making is affected by risk preference, self-control ability, and emotion, experimental data were collected using the Miners’ Risk Preference Scale, the Brief Self-Control Scale (BSCS), and the Positive and Negative Affect Schedule (PANAS).

##### Miners’ Risk Preference Scale

The risk preference index was evaluated using the 43-item Miners’ Risk Preference Scale prepared by Li et al.^42^. Risk is divided into cognitive preference, emotional risk preference, and three dimensions of behavioral intention preference, rated on a five-point Likert scale, from 5 (never occurred) to 1 (always is the case); the higher the score, the lower the willingness to take risks. Cronbach’s alpha was 0.93 in the current study.

##### BSCS

The BSCS, compiled by Tangney and Baumeisterv^43^ and revised by Tan and Guo^44^, was used as a screening tool for trait self-control. It consists of 13 questions divided into five dimensions, namely, overall self-discipline, impulse control, healthy habits, resistance to temptation, and reliability. The items are rated on a five-point Likert scale from 1 (completely inconsistent) to 5 (completely consistent). The higher the total score, the higher the level of individual trait self-control. Cronbach’s alpha was 0.79 in the current study.

##### PANAS

The Chinese version of Watson et al.’s^45^ PANAS was adopted. The scale has been observed to be consistent across cultures^46^; thus, it could be used to assess the emotional self-rating of Chinese miners. The scale is composed of 20 adjectives describing emotions, with participants asked to evaluate their emotions on a five-point Likert scale from 1 (almost none) to 5 (various). Positive and negative emotions are separated into two parts. A high score on positive emotion indicates that an individual is energetic and happy, while a low score indicates indifference. On the contrary, a high score on negative emotion indicates subjective confusion and a painful emotional state, while a low score indicates calmness. Cronbach’s alpha was 0.78 in the current study.

### Experimental procedures

For the selected participants, the experiment took place in a quiet training room in the Shouyang Coal Mine from July 28 to August 6, 2020. Participants were required to have obtained adequate rest and food the previous day and were not allowed to overeat, drink, or consume caffeine. After arriving in the training room, the participants first read and signed the informed consent form. They were informed that they would be paid for their participation in the form of 20% of the prize money obtained in the game. Afterward, they completed the questionnaire. Then, the participants attempted the BART and IGT on a Lenovo Desktop computer. After completing the task, they received 20% of the reward they earned in the experiment.

### Analysis process

SPSS 25.0 (IBM Corp, Armonk, NY, USA), Microsoft Excel 19.0, and Origin 2018 (OriginLab, USA) were used to analyze the data. A single factor analysis of variance (ANOVA) was conducted on demographic variables and emotion to exclude the influence of demographic variables and emotion on the mental fatigue effect. Regression analysis was conducted between additional variables and the BART value and net score to eliminate the interference and influence of non-research factors such as emotion, trait self-control, risk preference, and other additional variables on the experiment. The BART value and covariate and the net score and covariate of different mental fatigue degree were analyzed using repeated measurements variance.

### Ethics approval

This manuscript has not been published or presented elsewhere in part or in entirety.

### Consent to participate

All study participants provided informed consent.

## Results

### The pre-experiment analysis

**Figure 1:**
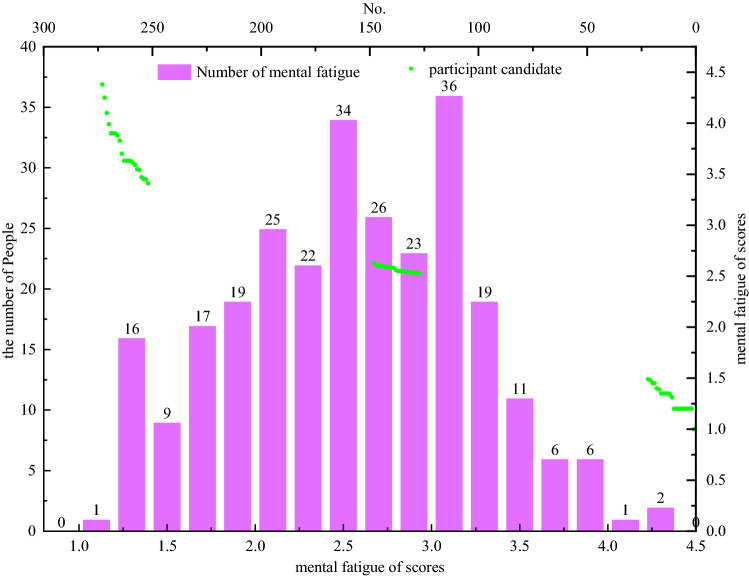
Miners’ mental fatigue scores and participant candidates’ statistical chart.

SPSS 25.0 (IBM Corp, Armonk, NY, USA) and Microsoft Excel 19.0 were used for the data analysis. We followed Kalimo et al.,33 who applied the weighted scoring method, that is, the score of mental fatigue = 0.40 × emotional exhaustion + 0.30 × dehumanization + 0.30 × low sense of achievement. The overall symptom score of mental fatigue is divided into mild, moderate, and severe. Through this analysis, we were able to select 22 participants with mild mental fatigue, 22 with moderate mental fatigue, and 22 with severe mental fatigue. Subsequently, we randomly selected 11 participants from these candidates according to their mental fatigue levels (11 participants were selected for each level of mental fatigue), enrolling 33 participants in the formal experiment. The remaining 33 participants took part in another experiment that ran simultaneously. The inclusion criteria were: regularly working underground in a coal mine, being right-handed, and having normal vision or corrected visual acuity (Fig. 1). After excluding three participants who were not serious about the experiment, we obtained experimental data for 30 participants.

### Descriptive statistical analysis of demographic variables

The 30 participants were male, right-handed, with normal or corrected visual acuity, and with no neurological disease. Participant ages were as follows: 10 participants were aged 24–30 years (33.33%), 16 were aged 31–40 (56.7%), and four were aged 41 or above (13.33%). Regarding educational level, 14 people had technical secondary school education or below (46.7%), 12 had junior college education (40%), and four had undergraduate degrees (13.3%). Regarding job role, there were 24 normal workers (80%) and six cadres (20%). Nineteen participants (63.3%) had worked for 0–5 years, nine had worked 6–10 years (30%), and two had worked for more than 11 years (6.7%). Regarding years of working underground, 21 people had worked for 0–5 years (70%) and nine for 6–10 years (30%).

Statistical analysis of participants’ demographic variables (Table 1) revealed no significant relationship between mental fatigue and age. However, the higher the education level and the greater the job responsibility, the higher the degree of mental fatigue. Further, increased mental fatigue was associated with more years worked and years of working underground. It showed that the effect of mental fatigue did not significantly differ by age, educational level, job role, and years of working. However, it significantly differed according to the number of years working underground. Therefore, the experience of the three groups (mild, moderate, and severe mental fatigue) was not significant in terms of demographic variables, such as age, educational level, job, working years, and birth in urban or rural areas, which undermines the influence of these factors on mental fatigue. However, the three groups of subjects differed significantly in terms of years working underground; thus, this variable was potentially related to mental fatigue. Further analysis was conducted of whether years working underground were correlated with the BART value and net score.

**Table 1:** Descriptive statistical analysis of demographic variables.

	Group	n	M	SD	SE	F	P
Age	Mild mental fatigue	10	36.20	6.30	1.99	1.33	0.28
	Moderate mental fatigue	10	31.90	5.49	1.73		
	Severe mental fatigue	10	32.90	6.69	2.12		
Educational level	Mild mental fatigue	10	1.40	0.70	0.22	1.97	0.16
	Moderate mental fatigue	10	1.60	0.70	0.22		
	Severe mental fatigue	10	2.00	0.67	0.21		
Job role	Mild mental fatigue	10	1.00	0.00	0.00	1.13	0.34
	Moderate mental fatigue	10	1.20	0.42	0.13		
	Severe mental fatigue	10	1.20	0.42	0.13		
Working for a fixed number of years	Mild mental fatigue	10	4.00	1.56	0.49	1.39	0.27
	Moderate mental fatigue	10	6.10	5.17	1.64		
	Severe mental fatigue	10	6.40	2.80	0.88		
Years of working underground	Mild mental fatigue	10	2.80	3.15	1.00	3.75	0.04
	Moderate mental fatigue	10	3.20	2.90	0.92		
	Severe mental fatigue	10	6.00	2.45	0.77		

M: mean; SD: standard deviation; SE: standard error.

### Analysis of emotion scale

The data gathered on emotions was meant to eliminate the interference of emotion as a factor. A single factor ANOVA test for positive mood showed that the mild mental fatigue group (mean [M] = 29.5, standard deviation [SD] = 6.311), moderate mental fatigue group (M = 31.3, SD = 7.47), and severe mental fatigue group (M = 29.1, SD = 8.40) showed neither significant positive emotional experiences, F (3, 30) = 0.248, P = 0.782, nor negative emotional experiences (M = 18.8, SD = 5.12; M = 23.8, SD = 3.65; M = 21.6, SD = 6.58, for each group, respectively). Thus, the influence of emotions on mental fatigue was excluded: F (3,30) = 2.274, P = 0.122.

### Analysis of BART values

#### Descriptive statistical analysis of BART values

**Figure 2:**
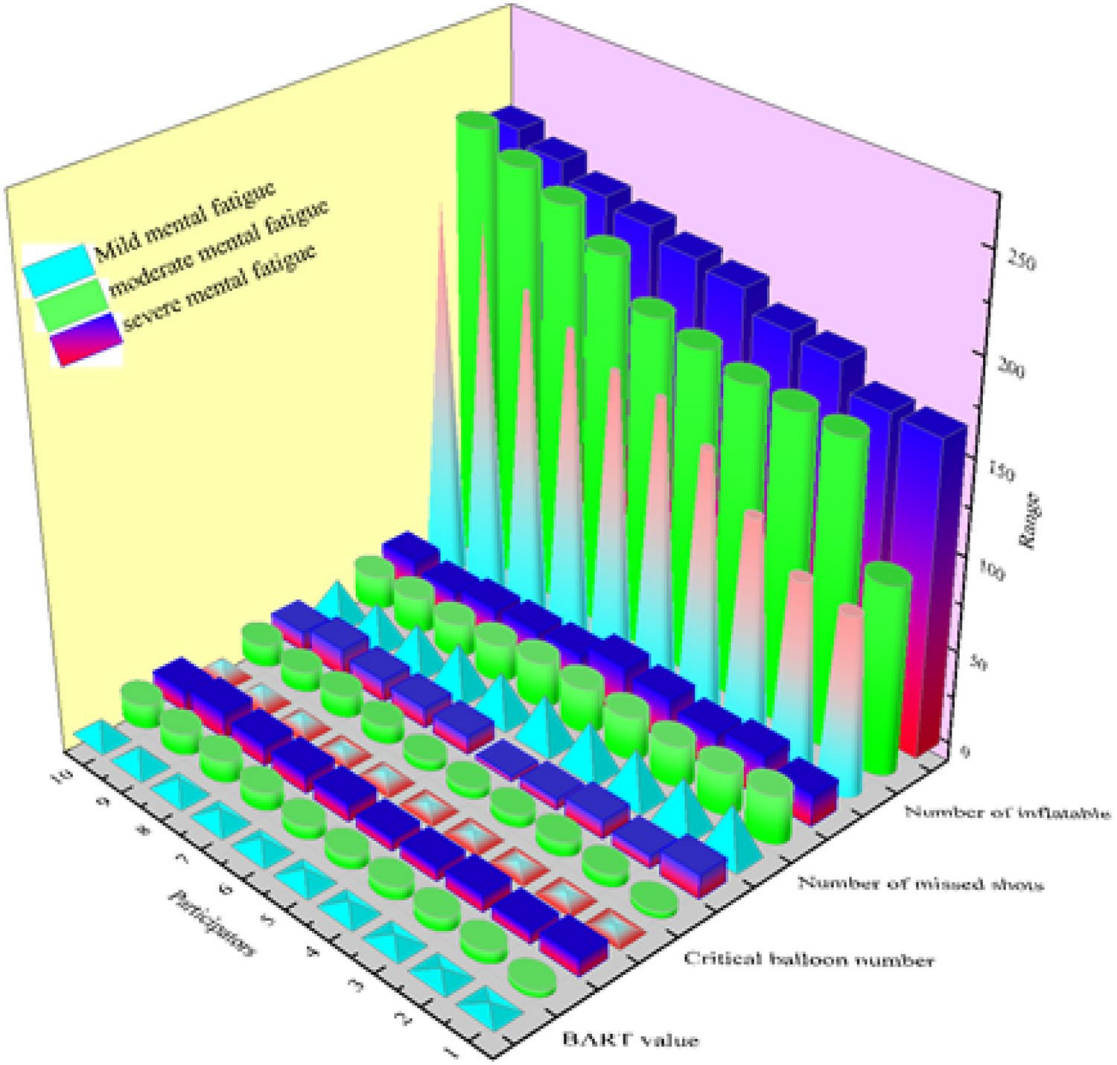
The results of correlation analysis between demographic variables and mental fatigue.

Figure 2 shows that with the increase of mental fatigue and the number of inflations and exploded balloons, the BART value gradually increases; the number of missed shots tends to decrease gradually. This suggests that with increased mental fatigue, the participants’ tendency to take risks is more obvious.

Descriptive statistical analysis of BART values for participants with mild, moderate, and severe mental fatigue including total number of inflations, total number of exploded balloons, total number of unexploded balloons, and BART values is exhibited in Table 2. The BART value is Mmild < Mmoderate < Msevere, that is, with the increase of mental fatigue, participants are more inclined to take risks.

**Table 2:** Descriptive statistical analysis of BART values.

	Group	N	M	SD
Total inflatable balloons	Mild mental fatigue	10	148.3	37.54
	Moderate mental fatigue	10	180.8	37.90
	Severe mental fatigue	10	196	20.31
Total exploded balloons	Mild mental fatigue	10	5.4	2.22
	Moderate mental fatigue	10	8.3	2.80
	Severe mental fatigue	10	9.8	3.55
Total unexploded balloons	Mild mental fatigue	10	24.6	2.22
	Moderate mental fatigue	10	21.7	2.80
	Severe mental fatigue	10	20.2	3.55
BART value	Mild mental fatigue	10	6.18	2.12
	Moderate mental fatigue	10	8.65	2.81
	Severe mental fatigue	10	10.02	2.34

#### Correlation analysis between additional variables and BART values

We conducted correlation analysis between the four factors and BART values to exclude the interference and influence of non-research factors such as emotion, risk preference, trait self-control, and underground working years. The results are shown in Table 3 positive emotion scores (R=-0.353, P<0.05), Risk preference (R=0.334, P<0.05), Trait self-control score (R=0.334, P<0.05) were significantly correlated with BART values. Therefore, for the BART, risk preference, trait self-control, and positive emotion score were incorporated into the equation as covariables for analysis (Table 3).

**Table 3:** Correlation analysis between additional variables and BART scores.

	1	2	3	4	5
1. Positive emotion score					
2. Negative emotion score	− 0.164*				
3. Risk willingness	− 0.226	0.474***			
4. Trait self-control score	− 0.161	0.216	0.073		
5. Years of working underground	0.049	− 0.076	0.234	− 0.275	
6. BART value	− 0.353***	0.093*	0.334***	0.39***	0.132*

***P < 0.001, *0.05 < *P* < 0.1.

#### Influence of mental fatigue on risk decision-making tendency

We found that BART values in the group with severe mental fatigue (M = 10.3, SD = 2.34) were greater than those in the moderate mental fatigue group (M = 8.65, SD = 2.81), which, in turn, were greater than those in the mild mental fatigue group (M = 6.19, SD = 2.12).

**Figure 3:**
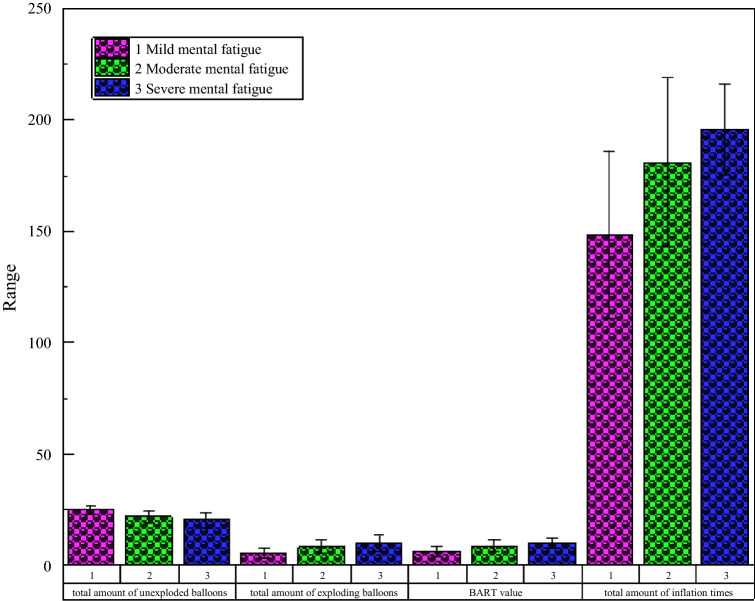
Statistical chart of BART experimental indicators.

To further study the effect of mental fatigue on risk propensity in decision-making, we performed an ANOVA with the mental fatigue groups as the independent variables; BART values as the dependent variable; and trait self-control, willingness to take risks, and working for a fixed number of years as covariates. The results showed that the difference in BART values between groups was significant: F (3, 30) = 4.142, P < 0.05. The bar chart (95% confidence interval) of BART values of different groups is shown in Fig. 3.

Overall, the results suggest that mental fatigue has a significant effect on risk propensity in risky decision-making. The higher the level of mental fatigue, the more risk-taking behaviors (the higher the reward) and the higher the risk-seeking tendency of the participants in the BART.

### IGT analysis

#### Number of card choices

**Figure 4:**
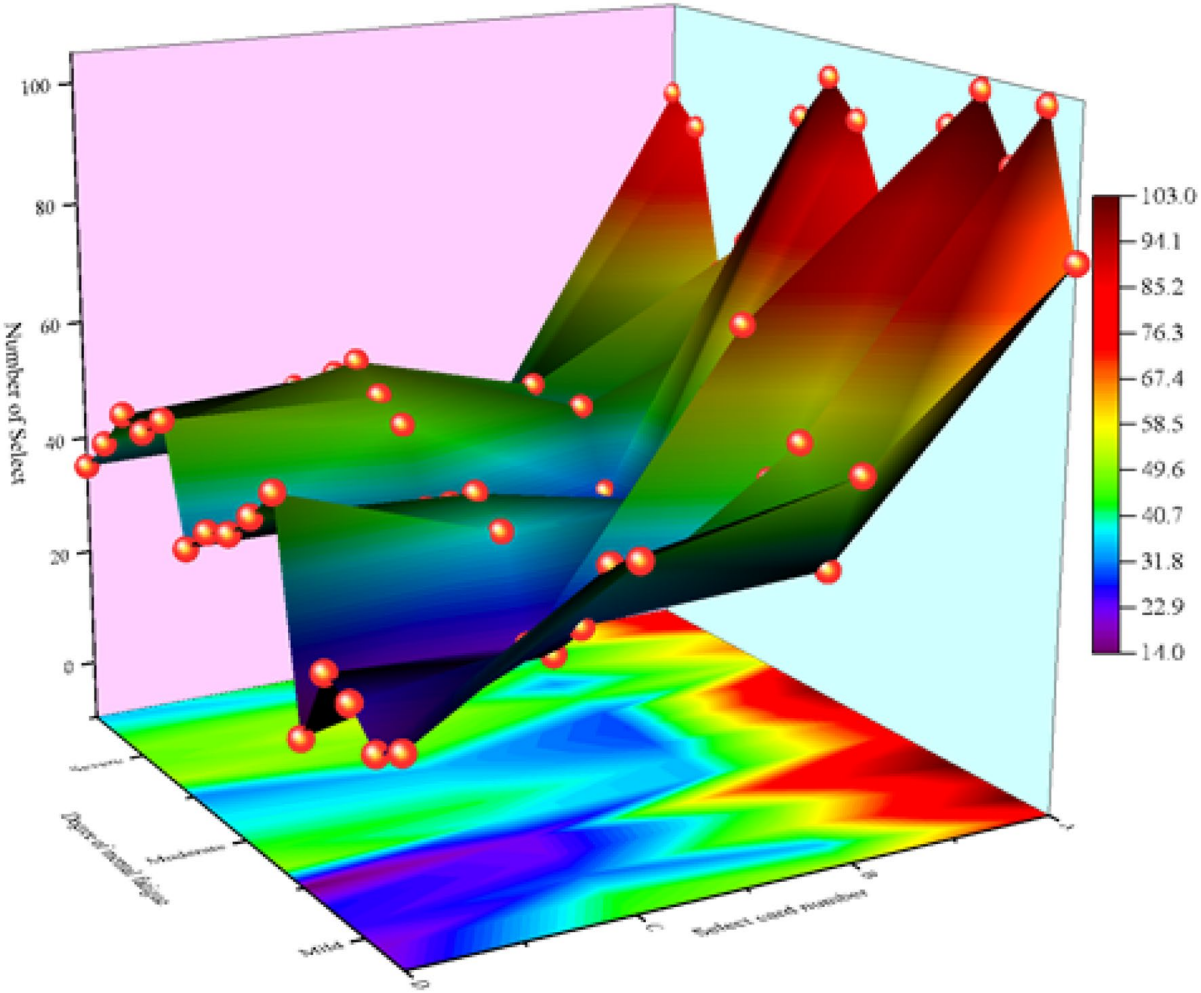
BART index evaluation in different mental fatigue groups (M ± SE).

In the IGT, high- and low-frequency reward decks contained low- and high-frequency penalty cards, respectively. Therefore, it was necessary to analyze the selection times of different types of cards by the three groups of participants to investigate their card selection characteristics. Figure 4 shows the selection times of different types of cards by participants with different degrees of mental fatigue. As shown, with a gradual increase in mental fatigue, the tendency to choose A/B cards decreased gradually, while the tendency to choose C/D cards increased gradually.

**Figure 5:**
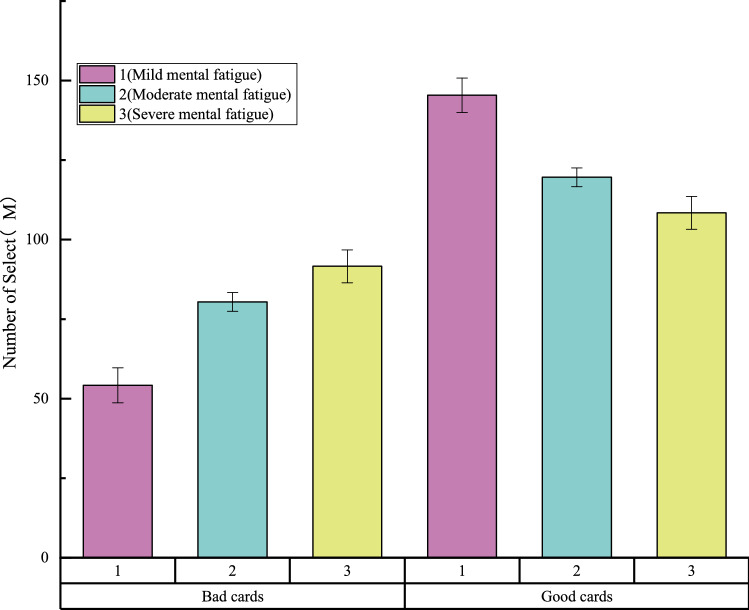
Number of cards selected for different levels of mental fatigue.

**Figure 6:**
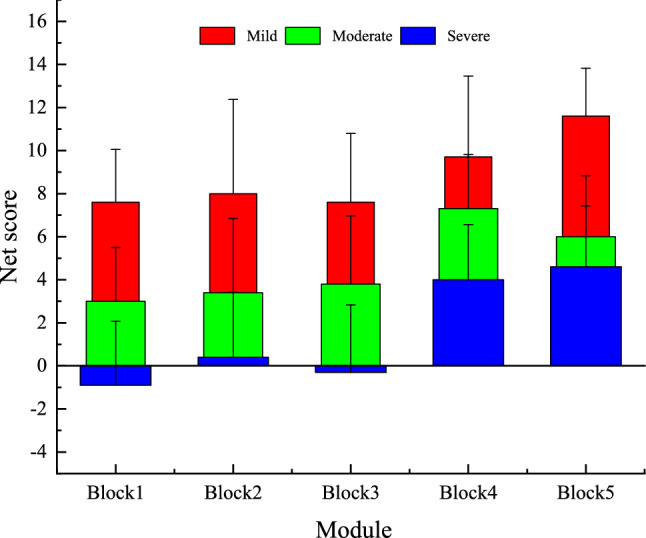
Statistical chart of favorable and unfavorable card selection (M ± SE).

Taking the type and class of cards as independent variables and the number of cards selected for each type as dependent variables, 2 (card type) × 3 (group) mixed-design ANOVA and ANOVA were carried out. The results showed that the main effect between favorable and unfavorable cards of card type was significant: F (2, 30) = 83.235, P < 0.001. The group main effect was also significant: F (3,30) = 16.789, P < 0.001. As shown in Fig. 6, with the aggravation of mental fatigue, the number of unfavorable cards gradually increased, while the number of favorable cards gradually decreased. Thus, with increasing mental fatigue, participants’ risk decision-making tendency became stronger. Further, the more serious the mental fatigue, the more likely they were to take risks (Fig. 5).

#### Statistical analysis of the net score of cards

A repeated measures ANOVA was performed with groups as the independent variables and the net score of each decision module as the dependent variable. The results are shown in Fig. 6. The main effect between modules was significant (F = 5.944, P < 0.05), but the main effect between groups was not (F = 2.43, P = 0.107 < 0.05). There was no significant interaction between the groups and modules: F = 0.177, P = 0.839. The net scores of the participants in the mild, moderate, and severe mental fatigue groups were significantly different, as shown in Fig. 6. The overall net score of the bar graph increases with the increase in decision times. Participants’ net score decreased with higher mental fatigue, reducing the overall height of the histogram. This indicates that as they experienced higher mental fatigue, participants became more inclined to take risks.

#### Correlation analysis between additional variables and net score

A correlation regression analysis was conducted between the four variables and the net score to exclude the influence of non-research variables such as emotions, trait self-control, risk preference, and years of underground work on risk decision-making. The results are shown in Table 4. Positive emotion score and net score (R = 0.296, P = 0.056) were significantly marginal. Net score had no significant correlations with negative emotion score, risk preference, trait self-control, or years working underground.

**Table 4:** Correlation analysis between additional variables and net score.

	1	2	3	4	5
1. Positive emotion score					
2. Negative emotion score	− 0.167*				
3. Risk willingness	− 0.233*	0.568***			
4. Trait self-control score	− 0.162*	0.315***	0.169*		
5. Years working underground	− 0.204*	0.131*	0.265*	0.13*	
6. Net score	0.296*	− 0.095*	− 0.238*	− 0.141*	− 0.138*

***P < 0.001, *0.05 < *P* < 0.1.

#### Influence of mental fatigue on risk decision-making tendency

The net score values of different groups, particularly, the net score value of the mild mental fatigue group (M = 44.5, SD = 40.43), were greater than those of the moderate mental fatigue group (M = 23.5, SD = 33.08) and the severe mental fatigue group (M = 7.8, SD = 38.2). The lower the net score, the more frequently the participants chose unfavorable cards and the greater their risk tendency, indicating that with higher mental fatigue, risk tendency increased.

To further study the effect of mental fatigue on risk propensity in decision-making, the mental fatigue groups were taken as the independent variables, with net scores as the dependent variables. The results of the regression analysis showed that the net score value differences between groups were significant: F (3, 30) = 4.992, P < 0.05. This indicates that mental fatigue had a significant impact on risk-taking tendency in risk decision-making; the higher the participants’ mental fatigue, the riskier their behaviors in the IGT (more frequently choosing unfavorable cards).

### Comprehensive risk score

The BART value and the IGT net score were tested using a paired samples t-test. The test results showed that T (30) = − 2.32, P < 0.05, F = − 0.283, P = 0.130. There was a very significant correlation between BART values and IGT net scores. These results indicate that the BART and IGT can be used to effectively measure risk-taking tendency and provide new ideas for workers’ job arrangements.

Taking mental fatigue as the control variable and BART values and IGT net scores as dependent variables, the correlation analysis showed that mental fatigue was negatively correlated with IGT net scores (F =  − 0.387, P < 0.05). In other words, the more serious the mental fatigue, the lower the IGT net score, the more frequently the participants chose unfavorable cards, and the greater the risk decision-making. There was also a significant positive correlation between mental fatigue and BART values (F = 0.543, P < 0.05). In other words, the more severe the mental fatigue, the higher the BART value, and the greater the risk decision-making tendency.

## Discussion

Previous studies have considered the effects of mental fatigue on risk-taking decisions. However, mental fatigue in all of these prior studies was transient mental fatigue, with the studies assessing people who were currently mentally fatigued. In contrast, very few studies have considered the impact of mental fatigue on risky decision-making, and there has been little research on the effect of mental fatigue on miners’ risky decision-making. This study focused on the effects of mental fatigue on risky decision-making among front-line miners. The results showed that the coal miners studied generally exhibited mental fatigue, although this was generally moderate; fewer miners exhibited mild and severe mental fatigue. In the BART experiment, the BART values of participants increased with increasing mental fatigue, indicating that decision-making was increasingly inclined toward risk-taking. In the IGT experiment, net scores decreased with increasing mental fatigue, which indicated that participants were increasingly inclined to take risks. The BART value had no or weak correlations with the net score. This is because, although the BART and IGT tasks examine the same risk-taking dimension, the IGT task has a strong learning effect, while the initial IGT score is very unstable. This results in a weak correlation between the two. In short, mental fatigue leads to an increased propensity to take risks.

In summary, increased mental fatigue was associated with an increasing tendency to make risky decisions. Therefore, it is important to regularly assess miners’ psychological fatigue and risk-taking tendencies, and to arrange work schedules accordingly. To ensure the safe operation of coal mines, it is important to allocate working time and intensity in reasonable manner, reduce or avoid night shifts, and ensure workers are provided rest time. These actions would reduce the occurrence of mental fatigue, and thereby minimize unnecessary accidents and losses caused by human behavior.

## Conclusion

With the increase of mental fatigue, BART value increased consistently, and participants were increasingly more inclined to take risks. In the IGT, with the increase of mental fatigue, the net score lowered accordingly, and the participants were increasingly inclined to choose from the unfavorable decks, that is, they were increasingly inclined to take risks. Based on these results, as mental fatigue increases, the risk tendency becomes increasingly stronger. The BART value had no or weak correlations with the net score.

In coal mine production, attention should be paid to workers’ mental fatigue, reasonable allocation of working hours, and working intensity. Avoiding the accumulation of mental fatigue by reducing or eliminating night shifts and ensuring that workers get adequate rest is important for avoiding accidents and losses.

## Data Availability

The data that support the findings of this study are available on request from the corresponding author, AJ. The data are not publicly available to ensure the privacy of the research participants.
